# Innovative fungal bioagents: producing siderophores, IAA, and HCN to support plants under salinity stress and combat microbial plant pathogens

**DOI:** 10.1186/s12934-025-02862-2

**Published:** 2025-11-25

**Authors:** Noura Al-Sissi, Mohamed H. Yassin, Radwan Khalil, Amina Gamal, Mohamed S. Attia, Amr H. Hashem

**Affiliations:** 1 Botany and Microbiology Department, Faculty of Science, Benha University, Benha, 13518 Egypt; 2 Botany and Microbiology Department, Faculty of Science, Al-Azhar University, Cairo, 11884 Egypt

**Keywords:** Plant growth-promoting fungi, Phosphate solubilization, Antagonistic activity, Salinity stress, *Triticum aestivum*

## Abstract

Salinity stress is a major environmental problem affecting agricultural productivity worldwide. Bioagents such as plant growth-promoting fungi (PGPF) are gained increasing attention to improve plant growth and resilience to this problem. This study addresses the isolation and screening of endophytic fungal isolates from *Atriplex nummularia* as well as soil fungi for salinity tolerance. Screening revealed two fungal isolates AS1 and B4, exhibiting exceptional salt tolerance at different concentrations of NaCl from 2 to 10%. Morphological and molecular identification confirmed AS1 was identified as *Alternaria* sp. and B4 as *Aspergillus terreus.* Results revealed that, both fungal strains are plant growth promoters under normal and saline conditions in vitro. In normal conditions, endophytic *Alternaria* sp. AS1 produced indole acetic acid (IAA) and solubilized phosphate with quantities 39.0 and 58.438 µg/ml; and *A. terreus* B4 with quantities 52.90 and 63.07 µg/ml respectively. In saline conditions, IAA production by both fungal strains was decreased gradually with increasing salt concentration. On the other hand, phosphates solubilization was increased with increasing salt concentration up to 8% where the quantity was 81.917 and 85.677 in the case of endophytic *Alternaria* sp. AS1 and *A. terreus* B4, respectively. Furthermore, both fungi produced siderophores and hydrogen cyanide, with *A. terreus* exhibiting high production under both normal and saline conditions compared to the endophytic *Alternaria* sp. AS1. Antagonistic assays revealed that both AS1 and B4 effectively inhibited the growth of fungal plant pathogens *Alternaria alternata* and *Fusarium oxysporum* using dual culture technique. Antimicrobial assay demonstrated significant efficacy of ethyl acetate extracts of both fungi against *A. alternata*,* F. oxysporum and Ralstonia solanacearum* using the agar well diffusion method. Furthermore, seed treatment with both fungal strains and their consortia alleviated the harmful effect of salinity stress and improved seedling growth parameters compared to untreated wheat seeds. Our findings suggest that endophytic *Alternaria* sp. and soil fungus *Aspergillus terreus* have potential as bio-inoculants to improve plant growth and its resilience in saline environments.

## Introduction

In agricultural systems, Plant growth and productivity are exposed to serious environmental stress, involving abiotic stress that is one of the biggest constraints on plant growth all over the world [1, 2]. Salinity stress is a critical abiotic challenge affecting global ecosystems, agriculture and food security worldwide. It causes a variety of agricultural problems, harms the physicochemical aspects of soil, restricts enzymatic and microbiological activity [3–6], and has detrimental effects on the growth, metabolism and production of many crops, particularly cereal crops. Wheat is considered a salt-sensitive plant and the principal crop in the world for food security [7–9].The excessive accumulation of toxic ions in saline soil affects seed germination, growth, and productivity of plants and also alters their morphological and biochemical activities by inducing osmotic potential, ion toxicity, metabolic disorders, and nutrient imbalances [10, 11]. The high concentrations of toxic ions of sodium chloride in the root zone can lead to a disruption of protein synthesis and photosynthetic processes by interfering with the nutrient’s absorption and growth regulators and increasing sodium ion Na^+^ assimilation, leading to Na^+^ accumulation in the cytoplasm and reducing the ratio of Na^+^/K^+^ that can trigger stress-tolerance genes [12, 13]. Moreover, salt ions frequently induce overproduction of reactive oxygen species (ROS) in plant tissues, which cases membrane injury by enzyme inactivation, lipid peroxidation, DNA damage, and protein oxidation in plants [14]. Also, the overproduction of ROS damages seeds germination and physiological and metabolic processes, resulting in cell death [15].

Due to the detrimental impact of salinity stress on plant growth, considerable efforts have been made to apply efficient and eco-friendly approaches, such as plant growth-promoting microorganisms (PGPMs), they have been effectively used to support agricultural sustainability and boost the tolerance of plants to abiotic and biotic stresses [16–18]. There are limited studies that have reported on the effect of salinity stress on fungal PGP activities and their efficacy in the induction of plants tolerance to salinity stress compared to plant growth-promoting bacteria. PGPF are fungi that either inhabit soil and plant rhizospheres or act as endophytic fungi that colonize plant tissues [19, 20], have been demonstrated to enhance growth and resistance of plants to various stresses [21, 22]. PGPF are an eco-friendly and effective alternative tool to the use of agrochemicals and shield plants from environmental stresses [23]. They have various direct and indirect mechanisms to enhance the growth of plant, including the production of siderophores, hydrogen cyanide and phytohormones, phosphate solubilization, and systemic resistance induction to environmental stresses [24]. Moreover, PGPF have an effective role in stimulating disease resistance and suppressing the invasion of phytopathogens by induction of systemic resistance, myco-parasitic and saprophytic resistance, root colonization [25], and antagonistic potential by competing for nutrients and space [26]. PGPF are able to ameliorate salinity stress through upregulating the activity of antioxidant enzymes, inhibiting Na^+^ uptake, improvement of root water uptake, reducing ethylene synthesis, mitigation of ionic imbalances, enhancing osmo-protectants biosynthesis, adjusting the ratio of Na^+^/K^+^ in the cell, production of growth hormones, and improving the efficiency of photosynthesis and water use [27–29]. Herein, this study aimed to isolate, identify, and evaluate salinity-tolerant plant growth-promoting fungi (PGPF) from *Atriplex nummularia* and soils, with a focus on assessing their biochemical activities, antagonistic potential against plant pathogens, and ability to enhance wheat seedling growth under salt stress, thereby exploring their potential as bio-inoculants for improving plant resilience in saline environments.

## Materials and methods

### Plant material

**Fig. 1:**
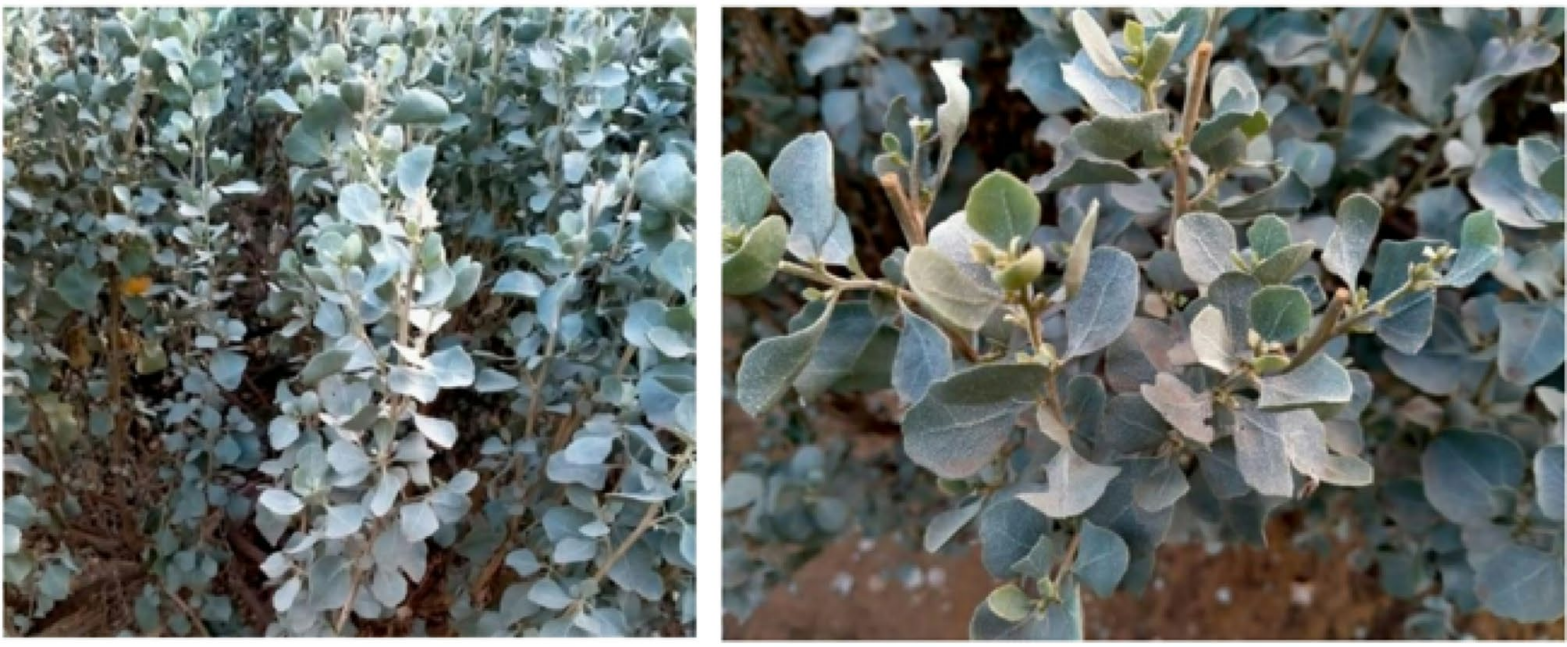
Photos of the collected *Atriplex nummularia*

Healthy plant samples of *Atriplex nummularia* were collected from different cultivated areas of Giza Park, Giza Governorate, Egypt (30° 04ʹ 28.2″ N and 31° 11ʹ 24.8″ E) (Fig. 1). The plant was taxonomically identified by Dr Saadia Hamed Aly at the Department of Botany and Microbiology, Faculty of Science, Benha University.

### Isolation of fungi

#### Isolation of endophytic fungi

Healthy plant samples were washed and dissected into stems and leaves. The plant parts were cut into segments and sterilized with 70% (v/v) ethyl alcohol for 2 min, 2.5% (v/v) sodium hypochlorite for 4 min, and following by washing with sterile distilled water three times [30, 31]. The dried and sterilized plant segments were inoculated on Potato Dextrose Agar (PDA) medium supplemented with 200 µg/l of chloramphenicol and incubated for 4–7 days at 28 ± 2 °C. The emerged colonies were purified and kept on PDA slants at 4 °C for further study [32].

#### Isolation of soil fungi

Samples of soil were gathered from different salt-affected areas in Wadi El Natrun Lakes, Beheira Governorate, Egypt (30° 24ʹ 29.1″ N and 30° 18ʹ 01.9″ E). Isolation was carried out using serial dilution of the collected soil sample. About 100 µl of serially diluted soil sample (10^−1^ to 10^−3^) were inoculated on PDA medium supplemented with 200 µg/L chloramphenicol [33, 34]. The remaining incubation and purification steps were carried out as described previously for endophytic fungi.

###  Salinity tolerance of fungal isolates

All fungal isolates were screened according to salinity tolerance. A disc of each fungal mycelium was inoculated on PDA supplemented with 0, 2, 4, 6, 8 and 10% sodium chloride individually [35]. Fungal isolates grown on PDA medium plates without sodium chloride served as a control. After incubation at the 7th day, the diameter of each mycelial colony was measured for each isolate, and the average of these measures was calculated [36, 37].

### Identification of the most potent isolates

The most potent isolates were identified morphologically based on macroscopic features such as the colony appearance, color and texture and their microscopic features. Moreover, the two fungal isolates were identified genetically based on the 28 S rRNA gene. The sequences of isolates were analyzed and compared with similar reference sequences retrieved from NCBI databases using the BLASTn tool. The phylogenetic and evolutionary analysis was conducted in MEGA 11 software using the Neighbour-Joining algorithm with bootstrap analysis.

### In vitro screening of fungal isolates for their potential PGP traits

#### Indole acetic acid (IAA) production

Potato dextrose broth (PDB) medium, enriched with varying concentrations of NaCl and L-tryptophan (0.1 g/l), was employed to assess indole-3-acetic acid (IAA) production. Fungal cultures were inoculated into the prepared medium and incubated in the dark at 150 rpm for 5 days [38, 39].

Following incubation, fungal mycelium and debris were removed through filtration. The resulting filtrate was then centrifuged, and the supernatant was combined with Salkowski’s reagent and incubated in the dark for 30 min [40]. The development of a pinkish-red hue indicated the presence of IAA. For quantitative analysis, the absorbance of the sample was measured at 530 nm, and the concentration of IAA produced was determined using a calibration curve [41].

#### Phosphate solubilizing activity

The phosphate growth medium from the National Botanical Research Institute (NBRIP) was utilized to assess soluble phosphate levels [42]. A volume of 500 µl of fungal cultures was inoculated into 50 mL of NBRIP broth medium supplemented with varying concentrations of NaCl. The inoculated cultures were incubated under shaking conditions for five days, while an uninoculated medium served as the control [41]. At the end of incubation, cultures were filtered and centrifuged at 4000 rpm for 30 min. The obtained supernatant was analyzed for soluble phosphate by the phosphomolybdate blue color method [43], where the sample absorbance was measured at 660 nm [44]. The phosphorus concentration was determined using the calibration curve of monopotassium phosphate (KH_2_PO_4_) [41, 45].

#### The production of hydrogen cyanide HCN

The HCN production was determined as described by the protocol of El-Rahman et al. [46]. Fungal isolates were inoculated in 125 ml glass vials containing 50 ml PD broth supplemented with 4.4 g/l of glycine, and non-inoculated glass vials were considered as control. Sterilized filter paper strips were dipped in an alkaline solution of picrate carbonate and then placed at the neck of the vials. Each vial was closed and sealed off with Parafilm and incubated at 28 ± 2 °C for 5 days. The color changes from yellow to brown or brick red indicated the isolate’s ability to produce hydrogen cyanide. The results were recorded as negative (−), low (+), moderate (++), or high (+++), respectively.

#### Siderophore production

Siderophore Production was determined by the ferric chloride FeCl_3_ test according to method used by Jalal and van der Helm [47]. Fungal culture grown in PDB was filtered, and the filtrate was centrifuged for 20 min. Then, 1 ml of the culture filtrate was combined with 2 ml of a 2% FeCl3 solution. Uninoculated PD broth was used as a control. The development of brown color indicated the production of hydroxamate in the culture fungal filtrate [38, 48].

### Antagonistic activity of the most potent fungal isolates against phytopathogenic fungi

The antagonistic activity of fungal isolates against *Alternaria alternata* and *Fusarium oxysporum* was screened by the dual-culture assay. The control plate was inoculated with a disc of tested pathogen mycelium at a distance of 1 cm away from the margin of the PDA plate, while the dual culture plate contained discs of the pathogen and fungal isolate on opposite edges in the PDA plate. After incubation, the inhibition percentage of pathogen growth was measured by the following equation:

$${\text{Percentage}}\;{\text{inhibition}}\;{\text{in}}\;{\text{mycelia}}\;{\text{growth}}=\frac{{A - B}}{A} \times 100$$

where A is the radius of the pathogen mycelia in the control plate, and B is the radius of the pathogen mycelia in the dual culture plate [49, 50].

### Evaluation of antimicrobial activity of the most potent fungal isolates

Two fungal isolates were inoculated separately in PDB and incubated for 15 days. The culture media was filtered, and the obtained filtrate was extracted with ethyl acetate. Then the upper organic layer was collected and evaporated using a vacuumed rotary evaporator at 40 °C. Finally, the crude extracts were collected and dissolved in 1% (v/v) dimethyl sulphoxide (DMSO) and kept at 4 °C [51–53]. The antimicrobial potential of ethyl acetate fungal extracts was evaluated against the bacterial plant pathogen (*R. solanacearum*) and fungal plant pathogens (*A. alternata* and *F. oxysporum*) by the well diffusion method. These phytopathogenic strains were obtained from the “Mycology research lab”, Botany and Microbiology Department, Faculty of Science, Al-Azhar University, Egypt. Inocula of fungal pathogens were spread separately on PDA medium and bacterial pathogen on nutrient agar medium. Wells were cut on each plate using a sterile tip, and each well was inoculated with 50 µl of different concentrations of each fungal extract (10, 5, 2.5 and 1.25 mg/mL), while the filled well with the DMSO acted as a negative control. The inoculated plates were kept at 4 °C for 2 h, then antifungal plates were incubated at 28 °C for 72 h, while antibacterial plates were incubated at 35 °C for 24 h. After incubation, antimicrobial activity was assessed by measuring the inhibition zone diameter (mm) [54–56].

### Evaluation of the effect of wheat seed treatment with PGPF under salinity stress

The impact of seed treatment with the fungal inducers was conducted under different salinity levels (0.00, 100, 150, 200, 250 and 300 mM). The wheat seeds of (*Triticum aestivum* cv. Giza 171) were surface sterilized with 2.5% (v/v) sodium hypochlorite for 2 min and washed five times with sterile distilled water [57]. Afterward, the surface sterilized seeds were treated with the culture filtrate (CF) of two fungal isolates separately and their mix by mixing 100 seeds with 50 ml CF, while the control seeds were soaked in 50 ml sterile distilled water. The fungal-treated seeds were incubated at 25 °C under shaking conditions for 5 h to facilitate the penetration of the fungal inducer. The presoaked seeds were placed in Petri dishes containing sterilized filter papers moistened with 10 ml sterile water or appropriate concentrations of NaCl (10 seeds per dish), then the plates were incubated in the dark for 10 days [58]. At the end of the experiment, plumule and radical length, and fresh and dry weights of plumule and radical were measured.

### Statistical analysis

The experiments in this study were conducted in triplicate and the obtained results were presented as mean ± standard errors. The obtained data were analyzed using the one-way ANOVA test and Duncan’s test at the 0.05 level. The data was statistically analyzed using SPSS software V. 14.

## Results and discussion

### Isolation of fungi and evaluating their salinity tolerance

A total of seven fungal isolates were isolated from *A. nummularia;* four fungal isolates from the leaf (AL1, AL2, AL3 & AL4) and three fungal isolates from the stem (AS1, AS2 & AS3). Also, five fungal isolates were isolated from the collected soil, where the codes were B1, B2, B3, B4 and B5.

**Fig. 2:**
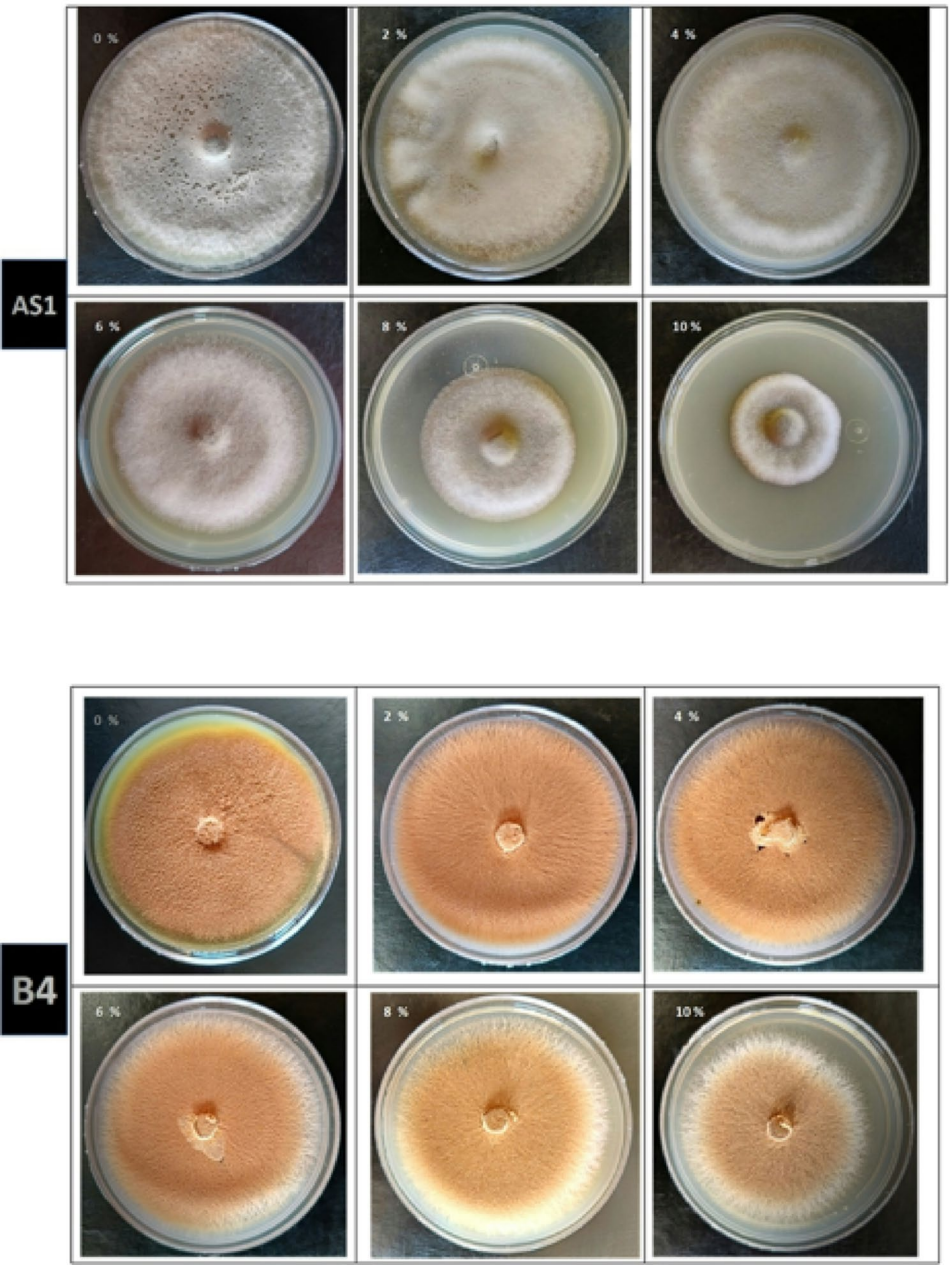
Fungal growth diameters of endophytic *Alternaria* sp. AS1 and *Aspergillus terreus* B4 on PDA with 0, 2, 4, 6, 8 and 10% sodium chloride

Then, all fungal isolates were screened according to their salinity tolerance, where each fungal isolate was grown on PDA supplemented with 0, 2, 4, 6, 8 and 10% sodium chloride. Results revealed that two fungal isolates AS1 & B4 out of the twelve fungal isolates have the highest tolerance to salt stress (Fig. 2). Fungal isolate AS1 could grow at all concentrations used in this experiment; the growth diameters at high concentrations of 8% & 10% were 55.89 ± 0.078 & 42.67 ± 0.038 mm, respectively (Table 1). As well, fungal isolate B4, which was isolated from the soil, has high tolerance to a concentration of 10% with a growth diameter of 69.56 ± 0.091 mm. Our findings align with Alhaddad et al. [50], Abdel-Ghany and Alawalqi [59] and Wang et al. [60], who showed the ability of *A.terreus* to tolerate stress and produce metabolites even to 10% NaCl. Moreover, Zhang et al. [61] highlighted the ability of endophytic *Alternaria* sp. 17,463 to ameliorate salt stress by regulating antioxidant enzyme production and also demonstrated that increasing salt stress induced radial growth inhibition of *Alternaria* sp. 17,463. In addition, Chauhan et al. [62] reported that salt-tolerant *A. niger* showed a significant ability to alleviate salt stress even at 15% NaCl, and Mahadik and Kumudini [63] highlight that *A. sojae* had the ability to grow under 1200 mM of salt stress. The fungal tolerance to salinity stress is explained by Badawy et al. [64], who reported that fungi are able to produce extracellular polysaccharides that cover and increase their cell wall thickness, also they form cell clumps to adapt to high salt stress [65]. Salinity tolerance by microorganisms could enhance the ability of plants to survive in harsh environmental conditions as salinity stress [50].

**Table 1:** The mycelial growth diameter of fungal isolates endophytic *Alternaria* sp. AS1 and *Aspergillus terreus* B4 on PDA with different salt concentrations

NaCl conc. (%)	Mycelial growth diameter (mm)	
	AS1	B4
0	80.00 ± 0.033^abc*^	80.33 ± 0.058^ab^
2	79.33 ± 0.033^bc^	81.56 ± 0.011^a^
4	76.78 ± 0.118^de^	79.33 ± 0.019^bc^
6	71.00 ± 0.088^f^	78.22 ± 0.022^cd^
8	55.89 ± 0.078^g^	76.22 ± 0.040^e^
10	42.67 ± 0.038^h^	69.56 ± 0.091^f^

* Letters a, b, c, … mean significance power

### Identification of fungal isolates AS1 and B4

**Fig. 3:**
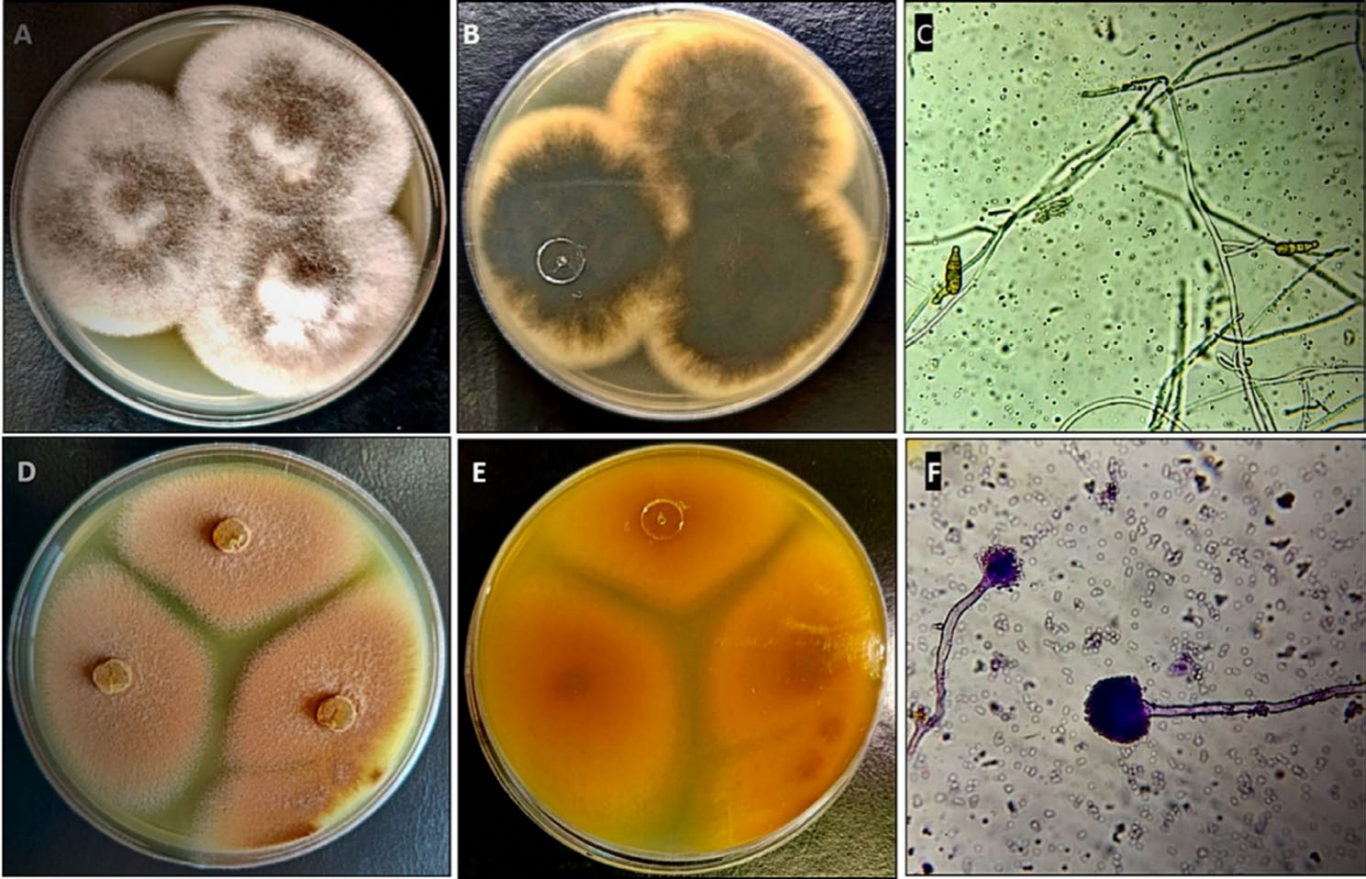
Morphological identification of fungal isolate endophytic *Alternaria* sp. AS1 (**A**–**C**): **A** Surface of the fungal colony; **B** Reverse color; **C** Conidia. Fungal isolate *Aspergillus terreus* B4 (**D**–**F**): **D** Surface of the fungal colony; **E** Reverse color; **F** Conidiophore & Conidia. Magnification power of microscopic photos is 400x

**Fig. 4:**
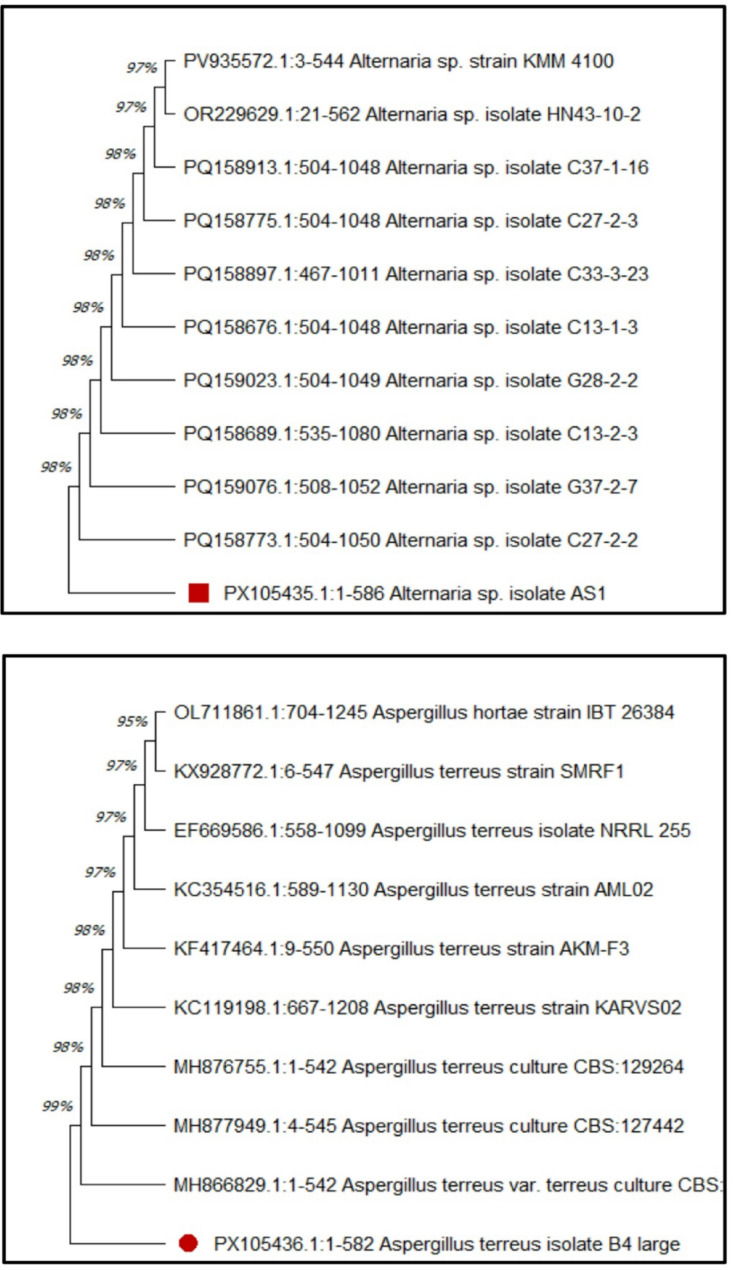
Phylogenetic tree of endophytic *Alternaria* sp. AS1 with accession number PX105435.1 (Above) and *Aspergillus terreus* B4 with accession number PX105436.1 (Below). No. of bootstraps = 500

The most potent fungal isolates, endophytic AS1 & soil B4, were identified morphologically based on appearance, color, and microscopic features. Morphological identification of the fungal isolate AS1 showed that the colony was grey woolly to cottony with a white margin, and the reverse of the colony was olive grey surrounded with a pale brown margin, as shown in Fig. 3A, B. Regarding microscopic examination, conidia are long ovoid with 1–4 transverse and 0–1 longitudinal septa (Fig. 3C). Moreover, morphological identification of the fungal isolate B4 displayed that the colonies had rapid growth with a powdery appearance. The colony surface is cinnamon to brown in color with yellowish-orange reverse (Fig. 3D, E). Microscopically, fungal isolate B4 showed hyaline and septated mycelium, non-septated and smooth conidiophores ending with globose vesicles bearing spherical and smooth conidia (Fig. 3F). Furthermore, the morphological identification was confirmed by molecular identification. The findings revealed that the fungal isolate AS1 was genetically identified as *Alternaria* sp. with 98% similarity, this isolate was deposited in GenBank with accession number PX105435.1 as *Alternaria* sp. Isolate AS1 (Fig. 4). Also, the fungal isolate B4 was genetically identified as *Aspergillus terreus* with 99% similarity, this isolate was deposited in GenBank with accession number PX105436.1 as *Aspergillus terreus* Isolate B4 (Fig. 4). Previous studies isolated and identified endophytic *Alternaria* spp. from various plants. Khalil et al. [66] isolated endophytic *Alternaria tenuissima* from leaves of *Avicennia marina*. Also, Elghaffar et al. [67] isolated *A. alternata* from *Ziziphus spina-christi* leaves that showed promising antimicrobial activities.

### Plant growth promotion capabilities of endophytic *Alternaria* sp. AS1 and *A. terreus* B4

#### Production of IAA

**Fig. 5:**
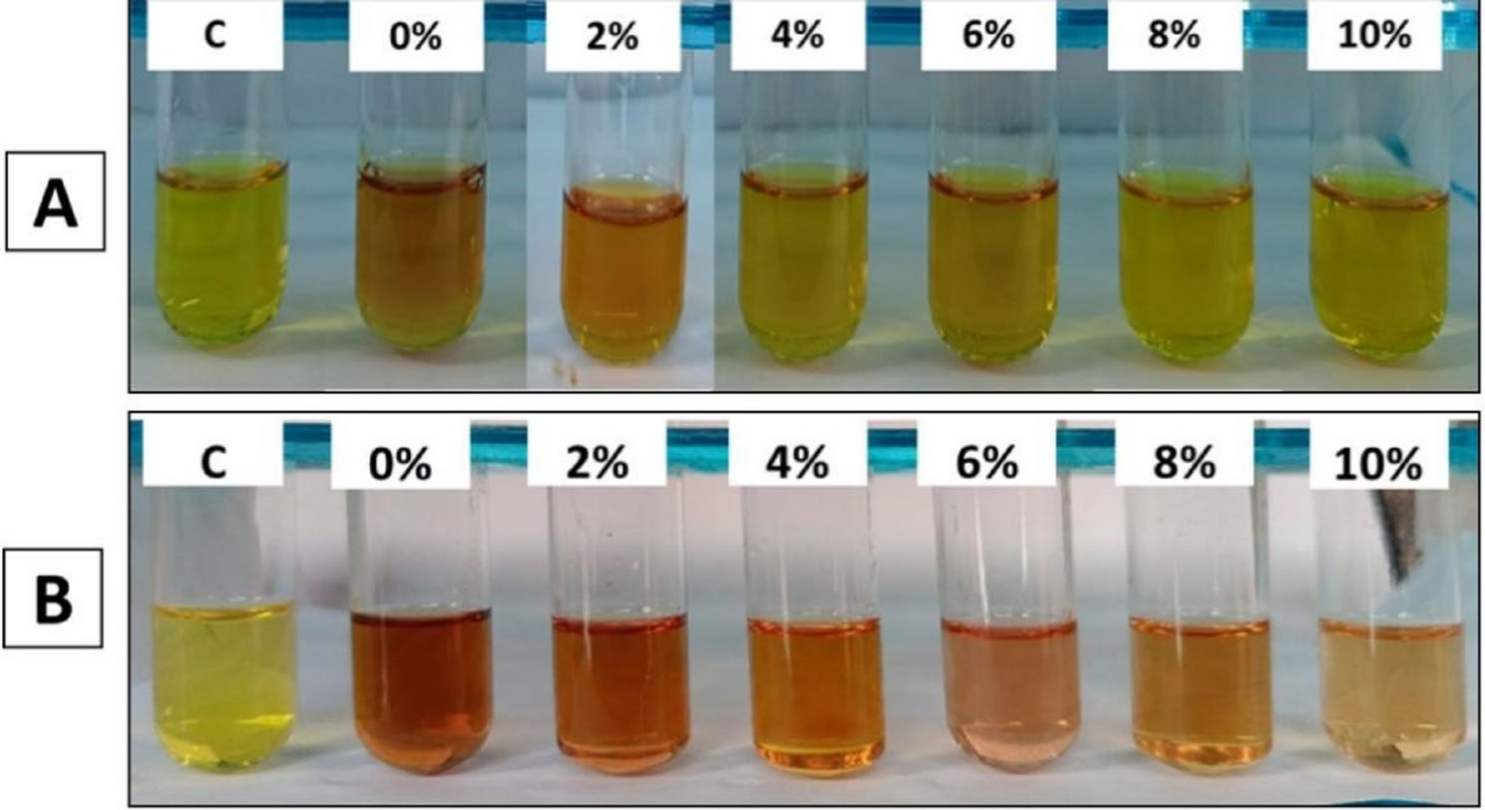
IAA production by endophytic *Alternaria* sp. AS1 (**A**) and *Aspergillus terreus* B4 (**B**) under normal and salinity stress conditions

The ability of fungi to produce phytohormones is crucial for enhancing plant growth. IAA is one of the crucial phytohormones that is responsible for cell growth, differentiating plant tissues, stimulating abscission, enhancing root elongation, and the formation of lateral roots and root hairs, which promotes the acquisition of nutrients and water by plants [68–71]. Besides improving plant growth, it also regulates the protective response of plants to abiotic stresses and enhances the interaction between plants and microbes [70, 72–74]. In the current study, endophytic *Alternaria* sp. AS1 and *A. terreus* B4 were evaluated for IAA production in normal medium and at different concentrations of NaCl (Fig. 5). Results revealed that both endophytic *Alternaria* sp. AS1 and *A. terreus* B4 have the ability to produce IAA under normal and stressed conditions. The IAA production was significantly influenced by increasing salinity levels. Under control conditions (0% NaCl), endophytic *Alternaria* sp. AS1 produced 39.005 µg/ml of IAA, while *A. terreus* B4 showed a marked highest production of 52.907 µg/ml. A gradual reduction in IAA production was observed with increasing in NaCl concentration in both fungi, as shown in Table 2. These results are consistent with Chaudhary et al. [75], who revealed that IAA production by *Metarhizium* strains was reduced at higher NaCl concentrations. The obtained results show that *A. terreus* B4 produced a higher amount of IAA under all salinity levels compared to endophytic *Alternaria* sp. AS1, indicating a good salt stress tolerance and its potential role in improving plant growth under salt stress. At a high concentration of 10% NaCl, the quantities of IAA produced from *A. terreus* B4 and endophytic *Alternaria* sp. AS1 were 27.796 and 9.507 µg/ml, respectively. These findings are consistent with Mahadik and Kumudini [63], Nusrat et al. [76] and Galeano et al. [77], who reported that fungi have the ability to release IAA under saline conditions.

**Table 2:** PGP traits of endophytic *Alternaria* sp. and *Aspergillus terreus* under different concentration of salt stress

Fungal strain	NaCl conc. (%)	Qualitative Analysis			Quantitative Analysis	
		IAA	HCN	Siderophore	IAA Conc. (µg/ml)	Phosphate Conc. (µg/ml)
Endophytic *Alternaria* sp. AS1	0	+++	+++	+++	39.006 ± 0.224^c^	58.438 ± 0.812^h^
	2	++	++	++	24.589 ± 0.338^g^	60.833 ± 0.227^g^
	4	+	+	++	20.090 ± 0.098^h^	67.708 ± 0.276^e^
	6	+	−	+	14.485 ± 0.161^i^	75.938 ± 0.502^d^
	8	+	−	+	14.006 ± 0.098^i^	87.917 ± 0.188^a^
	10	+	−	+	9.507 ± 0.516^j^	79.479 ± 0.188^c^
*A. terreus* B4	0	+++	+++	+++	52.907 ± 0.192^a^	63.073 ± 0.365^f^
	2	+++	+++	++	45.410 ± 0.354^b^	63.281 ± 1.365^f^
	4	+	+	++	32.922 ± 0.572^d^	74.688 ± 0.180^d^
	6	+	+	++	29.640 ± 0.098^e^	77.760 ± 0.454^c^
	8	+	−	++	28.829 ± 0.161^e^	85.677 ± 0.138^b^
	10	+	−	+	27.796 ± 0.064^f^	79.479 ± 0.998^c^

−, +, ++, +++ means negative, low, medium, high production respectively

Letters a, b, c, …. means significance power

#### Phosphate solubilization

**Fig. 6:**
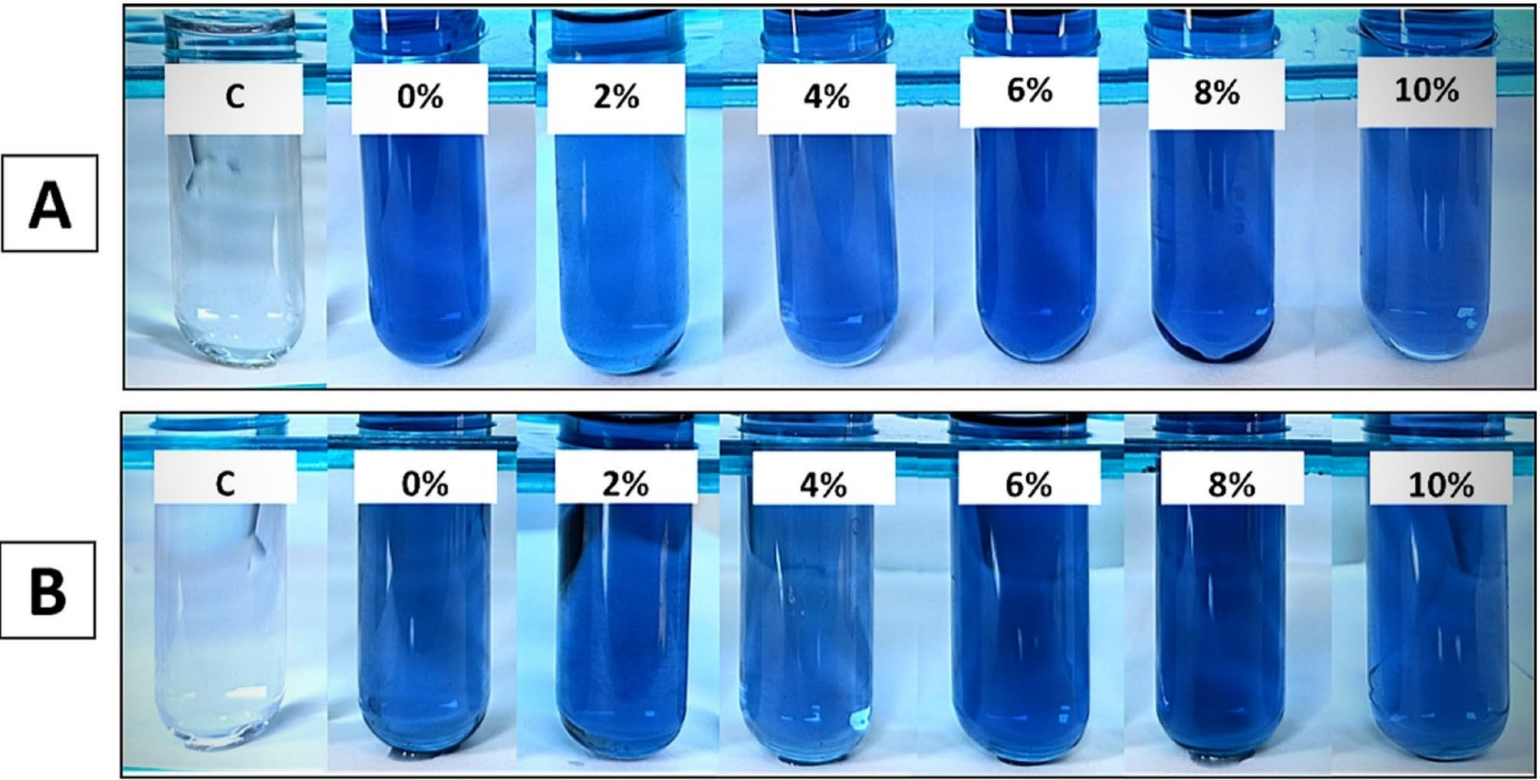
Phosphate solubilization by endophytic *Alternaria* sp. AS1 (**A**) and *Aspergillus terreus* B4 (**B**) under normal and salinity stress conditions

In the current study, the efficiency of endophytic *Alternaria* sp. AS1 and *A. terreus* B4 to solubilize phosphate under normal conditions and salinity stress was evaluated quantitatively by using NBRIP broth media (Fig. 6). Results illustrated that both endophytic *Alternaria* sp. AS1 and *A. terreus* B4 have the ability to solubilize phosphates under normal and salinity stress conditions. Also, the concentration of free soluble phosphate increased moderately with increasing salinity levels, as shown in Table 2. At a high salt level (8% NaCl), endophytic *Alternaria* sp. AS1 and *A. terreus* B4 exhibited the highest phosphate solubilization activity (87.916 and 85.677 µg/ml, respectively) compared to the normal condition. These observed results might be attributed to the ability of these fungal strains to synthesis suitable solutes and accumulate K^+^ ions to overcome the toxicity of Na^+^ ions as a response to salt stress [78]. Although previous studies have demonstrated that an increase in salinity causes a reduction in microbial activities, inhibition of various enzyme activities, disruption of membranes and protein denaturation [79], but some microorganisms have atypical membranes and produce salt stress proteins and synthesis osmolytes that eliminate the negative effects of salinity stress and allow them to live under such harsh conditions [80]. Our results are consistent with Galeano et al. [77] and Patel et al. [81], who reported that *Penicillium* sp. was able to solubilize phosphorus with higher concentrations in the presence of salt compared to the normal condition.

#### Siderophore production

**Fig. 7:**
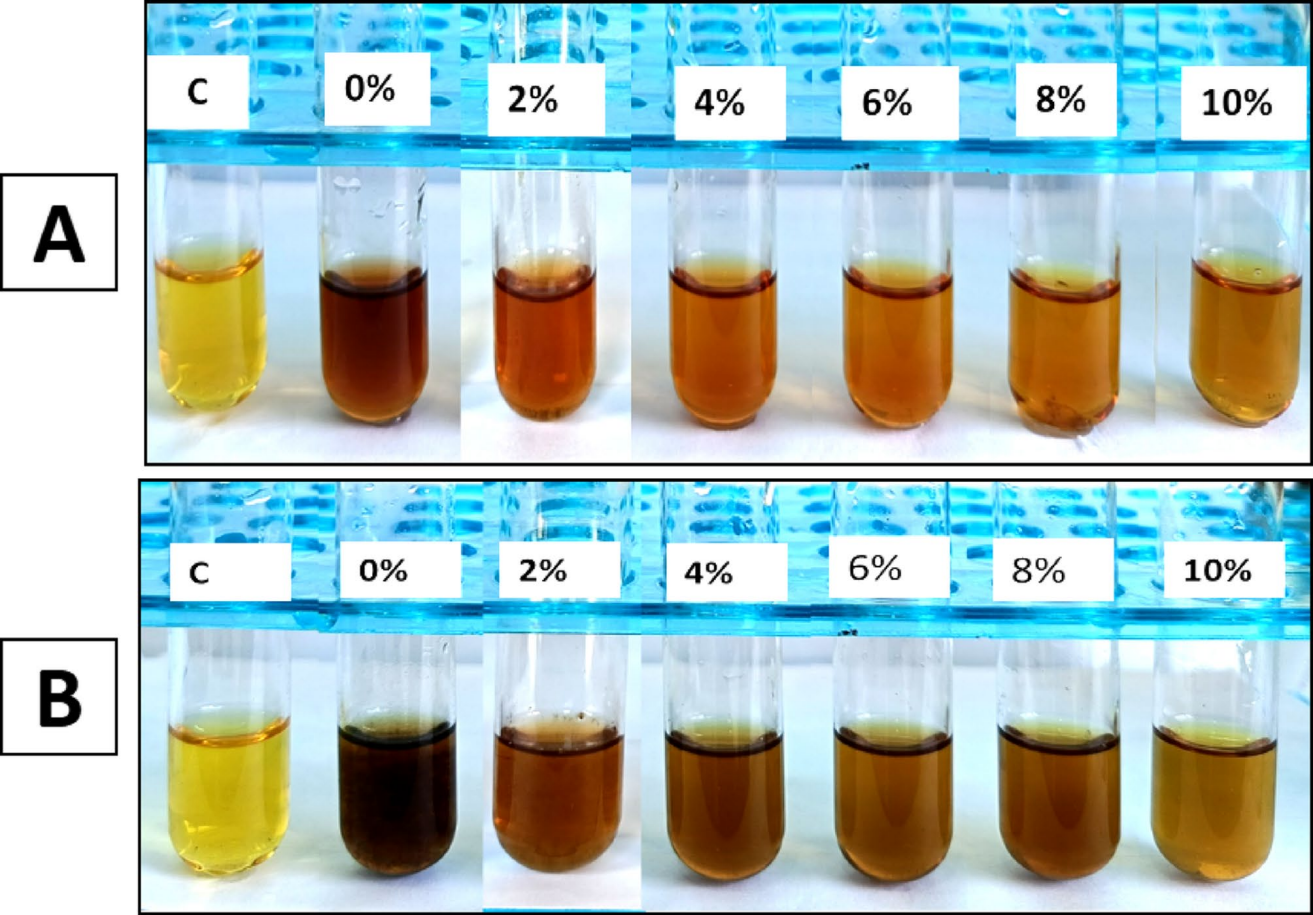
Siderophores production by endophytic *Alternaria* sp. AS1 (**A**) and *Aspergillus terreus* B4 (**B**) under normal and salinity stress conditions

Salinity stress is responsible for iron deficiency in plants, which can adversely affect plant growth and crop productivity. Siderophore-producing fungi are a promising and eco-friendly innovation for reducing iron deficiency and boosting plant growth in saline soils [82]. Fungi produce mostly hydroxamate-type siderophores, which are categorized into three families, including coprogens, fusarinines, and ferrichromes [83]. The production of hydroxamate-type siderophores in this study was qualitatively assessed using the ferric chloride test under varying salinity stress levels. A color change to reddish brown was observed under all salinity levels, indicating the production of hydroxamate-type siderophores. In the current study, both endophytic *Alternaria* sp. AS1 and *A. terreus* B4 showed changes in siderophore production under salinity stress, where the color intensity decreases gradually with increasing salt concentration. This finding is in line with [84], who found that increasing salinity levels resulted in the reduction of siderophore formation. As shown in Fig. 7; Table 2, A. *terreus* B4 showed the highest production under normal conditions and maintained moderate (++) production levels even at 8% NaCl. On the other hand, endophytic *Alternaria* sp. AS1 showed the lowest production at 4% to 10% NaCl. This finding is consistent with the studies that demonstrated siderophore production by *A. sojae* [63], *A. terreus* [85], and *A. chiangmaiensis* [86]. Additionally, siderophores exhibit a direct antagonistic role against plant pathogens, where they scavenge iron that is essential for pathogen growth and virulence, thus inhibiting their growth and indirectly improving plant growth [87]. Several studies reported the antimicrobial potential of fungal siderophores produced by *A. terreus*, *P. chrysogenum* and *A. sydowii* against phytopathogens that causing diseases in crop plants such as *Sclerotinia sclerotiorum*,* Ralstonia solanacearum* and *Xanthomonas oryzae* pv. *oryzae* also, bacterial siderophores were used in controlling the growth of phytopathogens such as *Pythium ultimum*,* Phytophthora parasitica and Sclerotinia sclerotiorum* [88–90].

#### HCN production

**Fig. 8:**
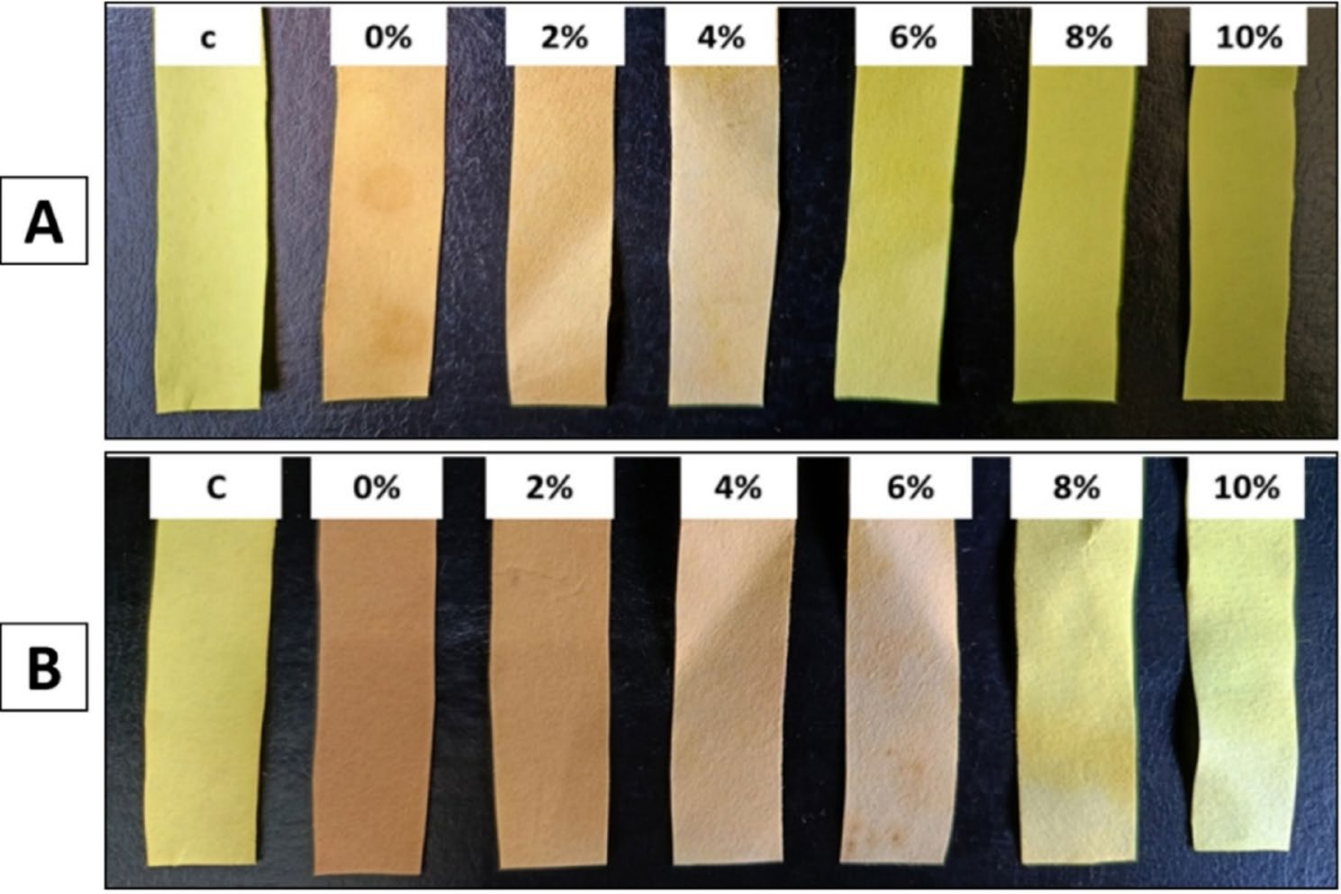
HCN production by endophytic *Alternaria* sp. AS1 (**A**) and *Aspergillus terreus* B4 (**B**) under normal and salinity stress conditions

In this study, the production of HCN under salinity stress was assessed based on the color intensity change of filter paper to reddish-brown. The results show a gradual decline in HCN production across different salinity stresses. The higher salinity levels (8%, 10%) were negatively affected and suppressed HCN synthesis in both fungal strains. As shown in Fig. 8, A. *terreus* B4 is more efficient in maintaining moderate HCN production under salt stress even at 6% NaCl compared to endophytic *Alternaria* sp. AS1, which showed low production under salinity stress with complete inhibition at 6% NaCl. This decline in HCN production by increasing salinity stress is consistent with the findings of [84], who reported that HCN production eventually decreased in different bacterial strains at 5% NaCl concentration.

### Antagonistic activity of endophytic *Alternaria* sp. AS1 and *A. terreus* B4 against phytopathogens using dual culture technique

**Fig. 9:**
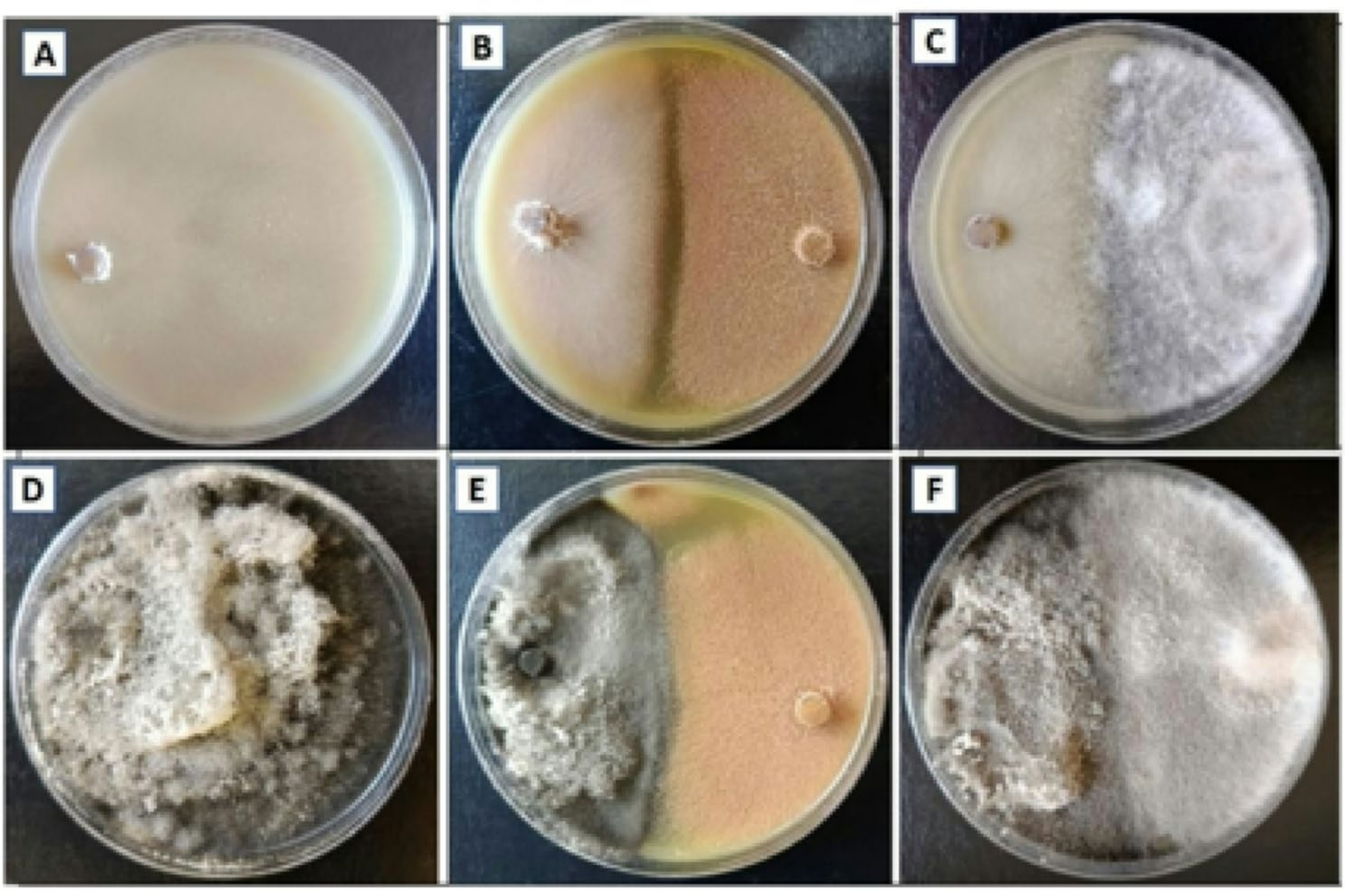
Antifungal activity using dual culture method: control *Fusarium oxysporum* (**A**), *Aspergillus terreus* B4 toward *Fusarium oxysporum* (**B**), endophytic *Alternaria* sp. AS1 toward *Fusarium oxysporum* (**C**), control *Alternaria alternata* (**A**) control *Alternaria alternata* (**D**), *Aspergillus terreus* B4 toward *Alternaria alternata* (**E**), endophytic *Alternaria* sp. AS1 toward *Alternaria alternata* (**F**)

The antagonistic effectiveness of two fungal isolates was performed using the dual culture assay against two phytopathogenic fungi, *F. oxysporum* and *A. alternata*, as shown in Fig. 9. The radial growth of phytopathogens was considerably reduced in the presence of the antagonistic fungi compared to the control plates. Results revealed that both *Alternaria* sp. AS1 and *A. terreus* B4 exhibited promising antagonistic activity against *A. alternata* and *F. oxysporum*. Figure 9B & E show the growth inhibition of *A. terreus* B4 against *A. alternata* and *F. oxysporum* was 58.53% and 62.47%, respectively, compared to the control (Fig. 9A). Likewise, endophytic *Alternaria* sp. AS1 exhibited inhibition toward both *A. alternata* and *F. oxysporum* with percentages of 55.49% and 64.9%, respectively, compared to the control (Fig. 9D).

### Antimicrobial activity of Ethyl acetate extract of endophytic *Alternaria* sp. AS1 and *A. terreus* B4 using the agar well diffusion method

**Fig. 10:**
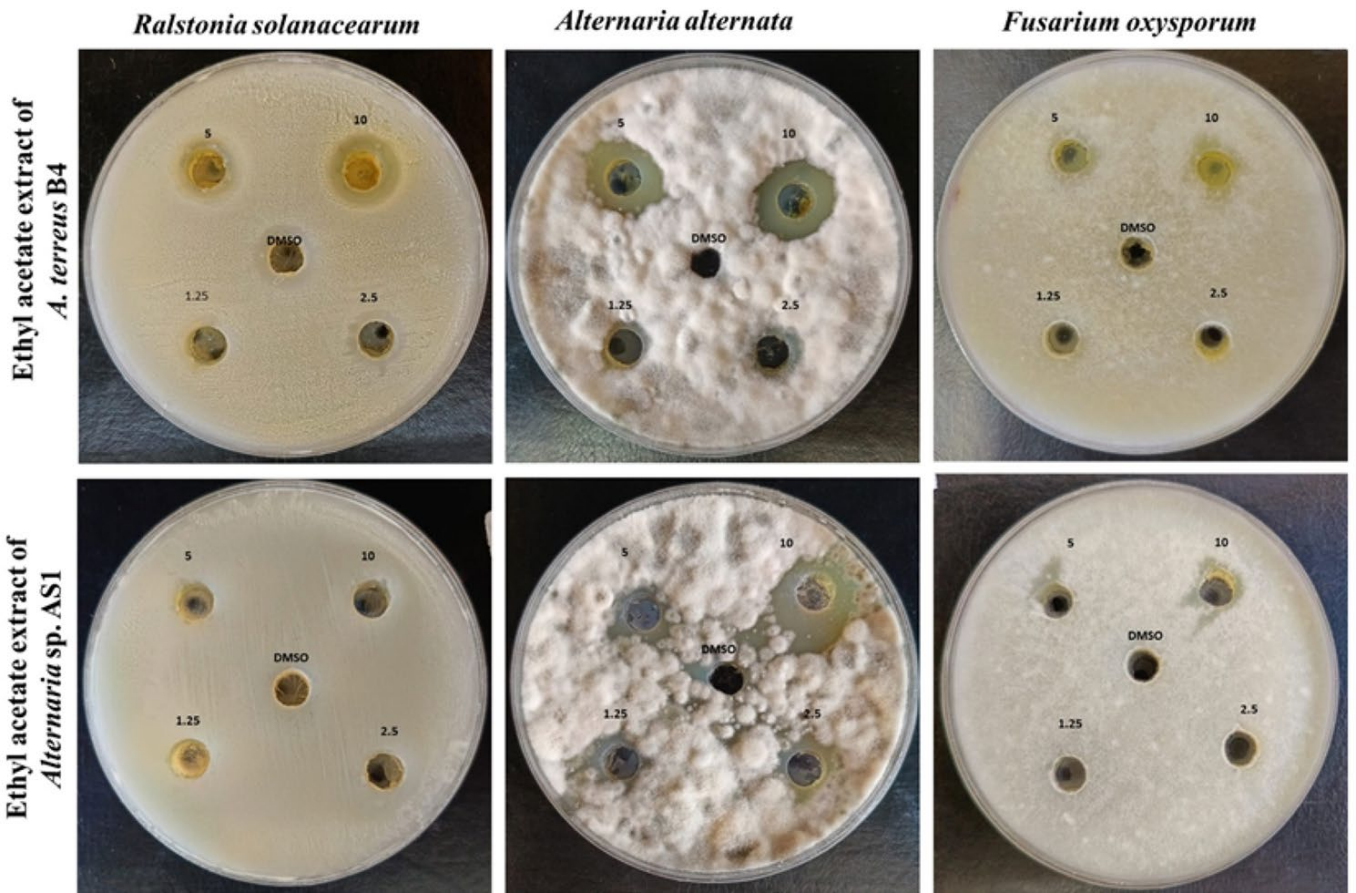
Antimicrobial activity of ethyl acetate extracts of endophytic *Alternaria* sp. AS1 and *Aspergillus terreus* B4 at different concentrations (1.25–10 mg/ml) against *Fusarium oxysporum*,* Alternaria alternata* and *Ralstonia solanacearum*

Antimicrobial activity of two ethyl acetate extracts of endophytic *Alternaria* sp. AS1 and *A. terreus* B4 was evaluated against the selected bacterial and fungal plant pathogens using the agar well diffusion method as shown in Fig. 10; Table 3. The results confirmed that the fungal extract of *A. terreus* B4 showed promising antifungal and antibacterial activity against *A. alternata*,* F. oxysporum* and *R. solanacearum.* Moreover, the highest efficacy of *A. terreus* B4 (10 mg/ml) was 19.56 ± 0.078 mm toward *A. alternata*, while the least efficacy with inhibition zone was 15.67 ± 0.0 mm toward *F. oxysporum* (Table 3). Furthermore, the ethyl acetate of endophytic *Alternaria* sp. AS1 showed antifungal activity toward *A. alternata* and *F. oxysporum*, where inhibition zones were 20.22 ± 0.109 and 12.44 ± 0.144 mm, respectively (Table 3). On the other hand, the ethyl acetate of endophytic *Alternaria* sp. AS1 did not show any activity at all used concentrations against *R. solanacearum.* The antagonistic effect of these fungi might be attributed to the production of plant growth stimulators and bioactive metabolites as proteins and enzymes that suppress the harmful effect of pathogenic microorganisms [91, 92]. Furthermore, these results can be explained due to the formation of HCN, siderophores, and IAA by fungi to enhance protection against phytopathogens, as reported by Abdelaziz et al. [93]. Our findings highlighted the antimicrobial activity of *A.terreus*, and these are consistent with many studies that reported the ability of *A. terreus* to produce novel metabolites that possess potential antimicrobial activities against the growth of *Pythium aphanidermatum* [94] and pathogenic bacteria [95]. In addition, endophytic *Alternaria sp.* are considered a promising source of bioactive compounds and used as biocontrol agents against pathogenic organisms and biostimulators for plant growth [92]. Elghaffar et al. [67] confirmed that *A. alternata* showed a promising antimicrobial potential against pathogenic bacteria and unicellular fungi. Moreover, Al Mousa et al. [96] stated that the ethyl acetate extract of *Alternaria tenuissima* had antimicrobial activity against *Fusarium solani* and *Aspergillus niger.*

**Table 3:** Antimicrobial activity of Ethyl acetate extracts of endophytic *Alternaria* sp. AS1 and *Aspergillus terreus* B4 against *Fusarium oxysporum*,* alternaria alternata* and *Ralstonia solanacearum*

Ethyle acetate extract	Phytopathogens	Growth-inhibition zone in (mm)			
		10 mg/mI	5 mg/ml	2.5 mg/ml	1.25 mg/ml
A. *terreus* B4	F. *oxysporum*	15.67 ± 0.0^a*^	12 ± 0.067^b^	NA**	NA
	*A. alternata*	19.56 ± 0.078^a^	16.33 ± 0.033^b^	13.11 ± 0.195^cd^	10 ± 0.033^e^
	R. *solanacearum*	17.33 ± 0.051^a^	15.00 ± 0.019^b^	12.22 ± 0.056^c^	NA
Endophytic *Alternaria* sp. AS1	F. *oxysporum*	12.44 ± 0.144^b^	11.11 ± 0.113^b^	NA	NA
	*A. alternata*	20.22 ± 0.109^a^	15.56 ± 0.089^bc^	12.78 ± 0.062^cde^	11.17 ± 0.010^de^
	S. *solanacearum*	NA	NA	NA	NA

* Letters a, b, c, …. mean significance power, Values are means ± standard error. & ** NA means no activity detected

### Phytotoxicity assay and evaluation of the effect of endophytic *Alternaria* sp. AS1 and *A. terreus* B4 inoculation on wheat seeds growth under normal and salinity stress conditions

**Fig. 11:**
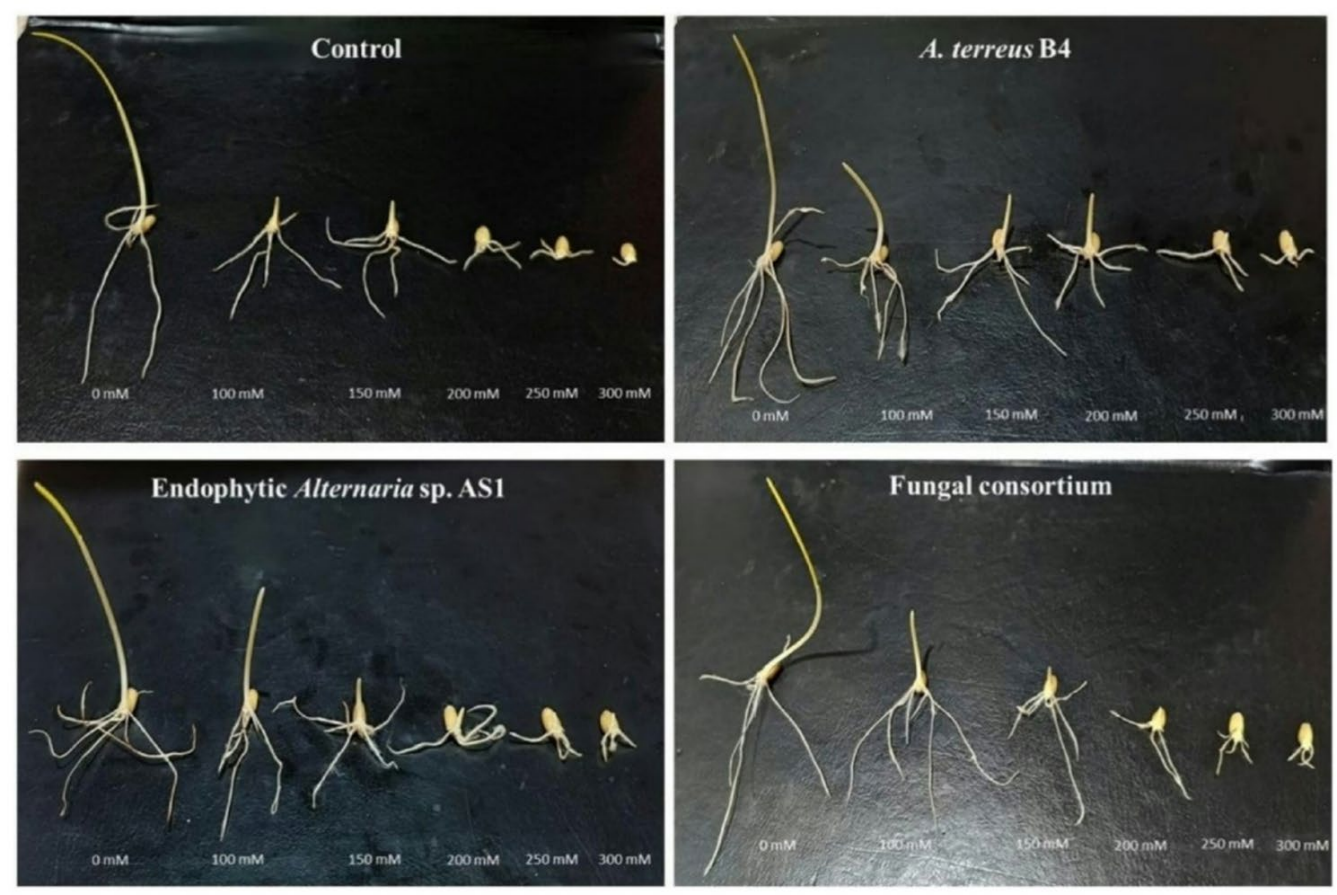
Effect of endophytic *Alternaria* sp. and *Aspergillus terreus* and their consortium inoculation on wheat seeds growth under different salinity stress (100–300 mM NaCl)

**Fig. 12:**
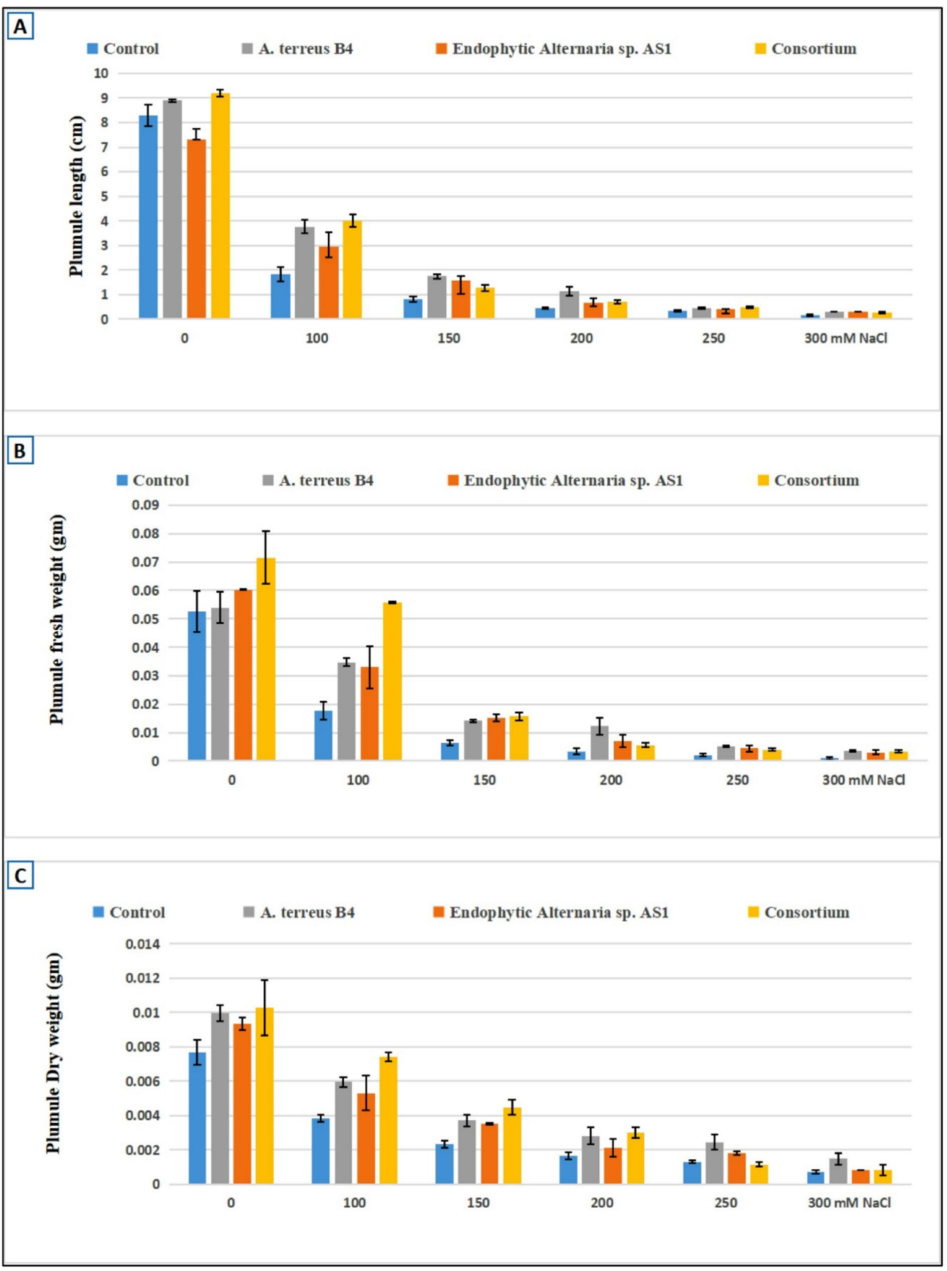
The effect of endophytic *Alternaria* sp. and *Aspergillus terreus* and their consortium on plumule length (**A**), fresh weight (**B**), dry weight (**C**) of wheat under different salt Conc. (100–300 mM NaCl)

**Fig. 13:**
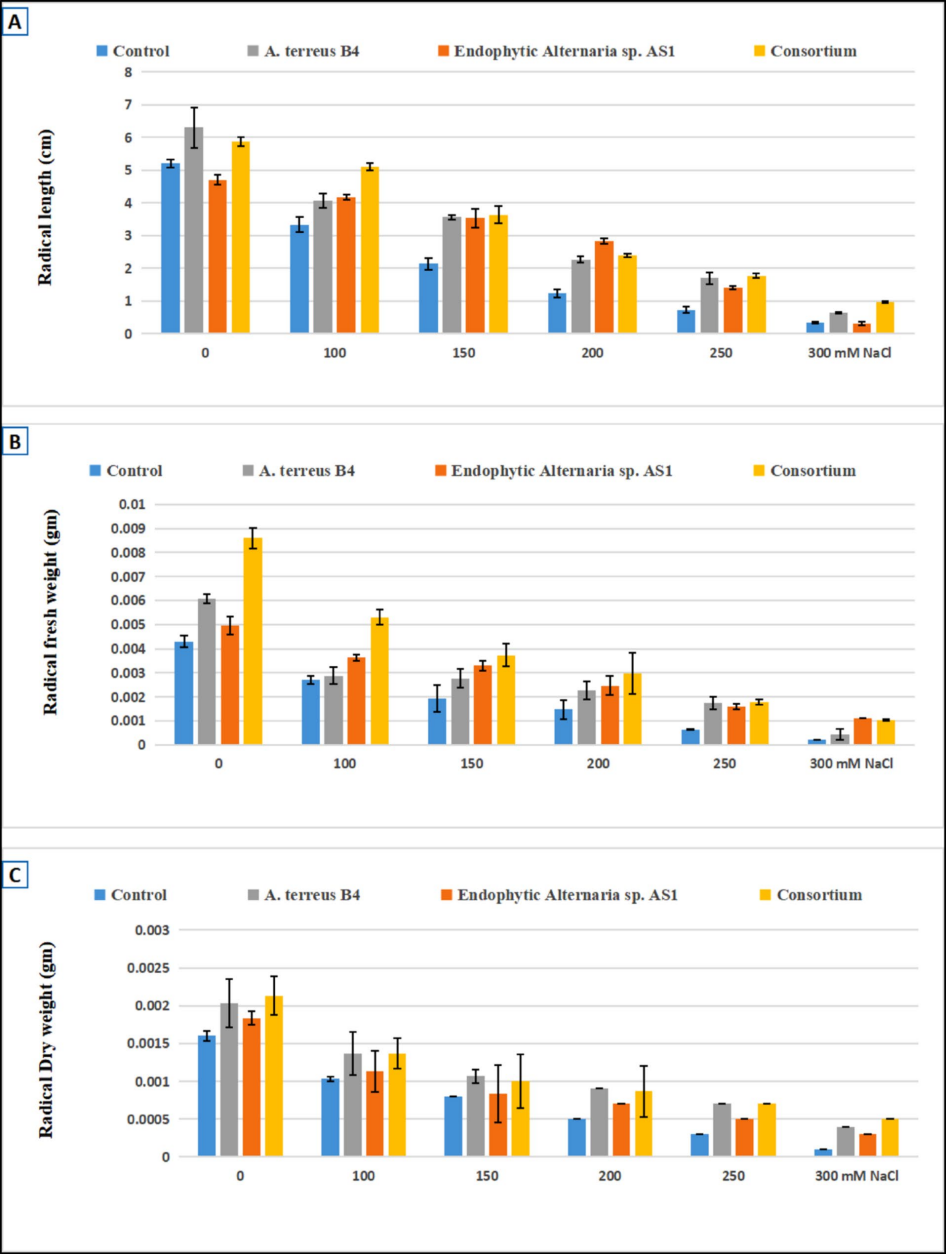
The effect of endophytic *Alternaria* sp. and *Aspergillus terreus* and their consortium on radical length (**A**), fresh weight (**B**), dry weight (C) of wheat under different salt Conc. (100–300 mM NaCl)

The phytotoxicity test is the first step to confirm the safety of new compounds or microbial extracts. Thus, in the current study, the phytotoxicity of endophytic *Alternaria* sp. AS1 and *A. terreus* B4 extracts was compared to the growth of wheat on distilled water. Results revealed that both fungal strain extracts have no cytotoxicity on the growth of wheat in vitro. Moreover, both fungal strain extracts increased the growth of wheat seedlings more than distilled water, as shown in Figs. 11, 12 and 13. These results confirm that both extracts of endophytic *Alternaria* sp. AS1 and *A. terreus* B4 are safe in use for agricultural applications.

Salinity stress has a negative impact on plant growth and causes a decline in the growth parameters as recorded in different investigations [97, 98]. Salt stress increasing in untreated plants reduced all morphological parameters of wheat plants. In contrast, in the current study, the application of endophytic *Alternaria* sp. AS1 and *A. terreus* B4 as bio-inoculants on wheat seeds alleviated the impact of salinity and enhanced seedling growth parameters (Figs. 11, 12 and 13). Across all salt stress, the seedling growth parameters were markedly decreased in untreated seeds compared to treated seeds with fungi, and this reduction may be due to the osmotic stress and ion toxicity resulting from salinity stress [99]. In the comparison with untreated wheat seeds at salinity stress, the application of endophytic *Alternaria* sp. AS1 and *A. terreus* B4 showed a significant increase in the length, fresh and dry weight of plumule and radicle. With respect to these results, Asaf et al. [100] found that treating soybean plants with *Aspergillus flavus* CHS1 increased the plant length, fresh and dry weight under normal and saline conditions. Moreover, the application of two fungal consortia showed a significant improvement in seedling growth parameters under normal and stress conditions (100 mM NaCl), and this effect might be the synergistic effect between two isolates that increase plant growth-promoting substances, which regulate and enhance plant growth under normal conditions or salinity stress [64]. These results are consistent with Anshu et al. [101], O’Callaghan et al. [102], and Muthuraja and Muthukumar [103], who reported that the synergistic effects of fungal consortia can improve plant growth and mitigate abiotic stresses.

## Conclusion

In conclusion, this study highlighted the significant role of bioagents, specifically plant growth-promoting fungi (PGPF), in enhancing agricultural resilience to salinity stress. Fungal isolates from *Atriplex nummularia and* soil samples were isolated and screened, leading to the identification of two promising candidates *Alternaria* sp. AS1 and *Aspergillus terreus* B4 that were found to demonstrate exceptional salt tolerance and beneficial traits such IAA, HCN, siderophore and phosphates solubilization. Moreover, IAA production was decreased under saline conditions, while as phosphates solubilization was increased, indicating adaptive resilience. Antagonistic assays revealed that both endophytic *Alternaria* sp. AS1 and *A.terreus* B4 effectively suppressed growth of *A. alternata* and *F. oxysporum* using dual culture technique. Furthermore, antimicrobial assays confirmed the potent antifungal activity of ethyl acetate extracts from both fungi. The efficacy of these fungi in alleviating salinity stress and promoting seedling growth was underscored, highlighting their potential as bio-inoculants in sustainable agriculture. This research has paved the way for further exploration of fungal bioagents to combat environmental challenges in crop production, contributing to global food security.

## Data Availability

The datasets analyzed during the current study are available in the NCBI GenBank database repository with the accession numbers of PX105435-PX105436.
